# Nitric Oxide-Induced Calcineurin A Mediates Antimicrobial Peptide Production Through the IMD Pathway

**DOI:** 10.3389/fimmu.2022.905419

**Published:** 2022-05-18

**Authors:** Kangkang Chen, Xinyan Wang, Xiangyi Wei, Jiaqian Chen, Youheng Wei, Haobo Jiang, Zhiqiang Lu, Congjing Feng

**Affiliations:** ^1^ Department of Plant Protection, College of Horticulture and Plant Protection, Yangzhou University, Yangzhou, China; ^2^ Department of Biotechnology, College of Bioscience and Biotechnology, Yangzhou University, Yangzhou, China; ^3^ Department of Entomology and Plant Pathology, Oklahoma State University, Stillwater, OK, United States; ^4^ Department of Entomology, College of Plant Protection, Northwest A&F University, Yangling, China

**Keywords:** insect immunity, reactive oxygen species, *Ostrinia furnacalis*, nitric oxide synthase, signal transduction

## Abstract

Nitric oxide (NO) at a high concentration is an effector to kill pathogens during insect immune responses, it also functions as a second messenger at a low concentration to regulate antimicrobial peptide (AMP) production in insects. *Drosophila* calcineurin subunit CanA1 is a ubiquitous serine/threonine protein phosphatase involved in NO-induced AMP production. However, it is unclear how NO regulates AMP expression. In this study, we used a lepidopteran pest *Ostrinia furnacalis* and *Drosophila* S2 cells to investigate how NO signaling affects the AMP production. Bacterial infections upregulated the transcription of *nitric oxide synthase 1*/*2* (*NOS1/2*), *CanA* and *AMP* genes and increased NO concentration in larval hemolymph. Inhibition of NOS or CanA activity reduced the survival of bacteria-infected *O. furnacalis*. NO donor increased NO level in plasma and upregulated the production of CanA and certain AMPs. In S2 cells, killed *Escherichia coli* induced *NOS* transcription and boosted NO production, whereas knockdown of *NOS* blocked the NO level increase caused by *E. coli*. As in *O. furnacalis* larvae, supplementation of the NO donor increased NO level in the culture medium and AMP expression in S2 cells. Suppression of the key pathway genes showed that the IMD (but not Toll) pathway was involved in the upregulation of *CecropinA1*, *Defensin*, *Diptericin*, and *Drosomycin* by killed *E. coli*. Knockdown of *NOS* also reduced the expression of *CanA1* and *AMPs* induced by *E. coli*, indicative of a role of NO in the *AMP* expression. Furthermore, *CanA1* RNA interference and inhibition of its phosphatase activity significantly reduced NO-induced *AMP* expression, and knockdown of *IMD* suppressed NO-induced *AMP* expression. Together, these results suggest that NO-induced AMP production is mediated by CanA1 *via* the IMD pathway.

## Introduction

Higher animals are armed with innate and adaptive immunity, but insects rely solely on less-specific innate immune responses to defend against invading pathogens in their habitats (1–3). In insects, bacterial and fungal pathogens trigger the host immune system *via* humoral and cellular components (4–6). Different immune challenges induce local and/or systemic responses (7, 8), suggesting that immune signaling pathways are extensively interlocked (9). Limited nutrients and short life spans for most insects require them to properly allocate energy between immune responses and other physiological processes such as development and reproduction (10–13). To maximize the reward of energy investment in immune responses, the interlocked immune signaling pathways must be regulated elaborately to avoid excessive immune responses. Reactive oxygen species (ROS) reaction and antimicrobial peptide (AMP) production are two primary humoral responses in the innate immune system of insects (4, 14). Insights into the cross-talk between them are important for understanding how different defense responses are coordinated to control infections.

ROS formation is a rapid, early response to pathogen invasion in insects. ROS can directly kill the invaders as effectors or function as signaling molecules to regulate the immune responses (15, 16). On the other hand, ROS can damage host cells as well (17). ROS include superoxide anion ${\text{O}}_{2}^{•-}$ , H_2_O_2_, OH^•^, ^1^O_2_, and NO, each with a highly reactive oxygen atom (1, 18–20). They act as signaling compounds and/or toxic byproducts in cells (21). Among them, NO is a gaseous free radical functioning as a signal messenger for several physiological processes (22), including regulation of innate immunity (23–25). In mammals, NO is produced by nitric oxide synthase-2 (NOS2) in macrophages to control bacterial infection, which induces *NOS2* transcription (26). In *Drosophila melanogaster*, NO is involved in the hemocyte encapsulation (27). In mosquitos, NO kills the *Plasmodium* parasites and increased NO to control the infection. Inhibition of NOS increases the rate of *Plasmodium* infection and that results in more deaths of infected mosquitos (28, 29). Blood meal taken by *Anopheles stephensi* catalyzes the conversion of NO to toxic metabolites, which kill the parasite in the gut (30).

AMP production is an effective immune response against microbial infection in insects (31, 32). They kill bacteria, fungi, and viruses sometimes (33–36). The Toll and IMD pathways actively participate in AMP production. Peptidoglycans (PGs) in the bacterial cell wall are recognized by the peptidoglycan recognition protein (PGRPs) to trigger the Toll and IMD pathways directly or indirectly. DAP-PGs from Gram- and some Gram+ bacteria are recognized by PGRP-LC/LE to induce the processing of IMD, FADD, Dredd, and Relish, and then the cleaved Relish enters the nucleus to activate AMP transcription (14, 36–40). Proteolytically activated Spätzle binds to the transmembrane receptor Toll to induce the intracellular signal transduction through MyD88, Tube/Pelle and Cactus. Finally, transcription factors such as Dorsal and Dif translocate into the nucleus to trigger AMP expression (14, 36, 38–40). Besides the classical Toll and IMD pathways, NO, eicosanoids, and calcineurin are also involved in the induced synthesis of AMPs in several model insects (25, 41).

Cross-talks among immune pathways keep the insect defense system running effectively and economically (15, 16, 42). Innate immunity is conserved at different levels in mammals, insects, and plants (43–45). In *Drosophila* larvae, NO activates the IMD pathway to produce Diptericin after infection by Gram-negative bacteria (46). The ROS stress upregulates NO production to enhance Diptericin synthesis in the adult gut (15). Furthermore, calcineurin subunit CanA1 is required for the NO regulation of AMP production in the fly (47). In *Spodoptera exigua*, injection of NOS inhibitor or knockdown of *NOS* reduced the AMP expression. In the absence of bacteria, an NO analog induced AMP expression (25). A cytokine named paralytic peptide induced NOS expression in the silkworm and triggered the AMP transcription in fat body (48). While AMP induction by NO is independent of the IMD or Toll pathway in *Drosophila* (41), this is dissimilar to the case in *S. exigua* (25), indicating that mechanism for NO regulation of AMP expression is unclear in insects. Comparative studies in different species are therefore needed to understand reasons for the discrepancy. Towards this goal, we used the Asia corn borer *Ostrinia furnacalis* as a model to investigate how NO signaling may communicate with the signaling pathways for AMP induction. NO strongly upregulated the AMP expression in *O. furnacalis* larvae, inhibition of NOS or CanA caused higher susceptibility of *O. furnacalis* to bacterial infection. In *Drosophila* S2 cells, IMD pathway connects NO signal to AMP production through CanA1.

## Materials and Methods

### Cell Culture and Insect Rearing

*Drosophila* S2 cells (Thermo Fisher, R69007) were maintained in Schneider’s *Drosophila* medium (Merck, S9895) containing 10% fetal bovine serum (FBS, Thermo Fisher, A3160802) (49). S2 cells were cultured in a 27°C incubator. All the S2 cells were plated in 12-well plates at 1×10^6^ cells/well for different treatments (1 mL medium per well). Asian corn borers, *O. furnacalis* larvae were reared using an artificial diet at 25 ± 1°C, RH > 80%, and with a photoperiod of 16 h light and 8 h darkness (13, 50).

### Bacterial Culture and Preparation of Dead Bacteria

Wild-type bacteria *Escherichia coli*, *Pseudomonas aeruginosa* and *Micrococcus luteus* (All the bacteria strains were kindly donated by Professor Zhiqiang Lu, Department of Entomology, College of Plant Protection, Northwest A & F University, China) picked from LB plates were grown overnight in Luria-Bertani (LB) medium at 37°C and 200 rpm. The 100 μL cultured bacteria were then inoculated into 10 mL fresh LB medium and cultured at 37°C until OD_600_ was close to 0.6. Finally, the bacteria were harvested by centrifugation at 8000×*g* for 10 min. After washing for 3 times, bacteria pellets were resuspended with phosphate buffered saline (PBS) for injection. To prepare the dead bacteria, *E. coli* and *M. luteus* cells from 100 mL LB medium were resuspended in 1 mL PBS and 40 mL 75% 2-propanol. After incubation for 1 h at 37°C and 200 rpm, dead bacteria were spun down and washed 3 times with PBS. Finally, the dead bacteria were resuspended in 1 mL PBS to treat S2 cells.

### Survival Rate Assay of *O. furnacalis* Larvae after Infection

To determine the number of bacteria for injection, day 1, 4^th^ instar *O. furnacalis* larvae were fed on artificial diet containing 5 μL (10 μg/μL) tetracycline that eliminates indigenous bacteria. The diet was replaced with fresh diet without antibiotic at 24 h post antibiotic treatment. Day 3, 4^th^ instar larvae were injected with 1×10^3^, 1×10^4^, 1×10^5^, or 1×10^6^ live cells of *P. aeruginosa* or *M. luteus*. PBS was used as control. There were 20 larvae in each group. The survival in each group was recorded at 12 h intervals. All the data was analyzed by the log-rank test using Prism 5.0.

### Treatment of S2 Cells and Infection of *O. furnacalis* Larvae

S2 cells cultured in 1 mL medium were incubated with 20 µL of killed bacteria at different amounts. At 24 h post bacterial exposure, the medium was collected for nitric oxide determination, and 500 µL Trizol (Invitrogen) was used to extract RNA from the S2 cells for qPCR analysis. Day 1, 4^th^ instar larvae were fed on the diet containing 50 μg/μL tetracycline to eliminate indigenous bacteria before injection with bacteria as described previously (51). At 24 h after antibiotic feeding, larvae were transferred to fresh diet without antibiotic. Day 3, 4^th^ instar larvae were injected with 1×10^4^ of live *P. aeruginosa* and *M. luteus* or along with NOS inhibitor/CanA inhibitor (2 nmol each) for determination of survival curve or qPCR analysis. All the results generated in survival assay were recorded at 12 h intervals and the whole *O. furnacalis* larvae at certain times post infection were treated with Trizol regent for RNA extraction (Invitrogen). PBS was used as control. All the treatments were performed in triplicate.

### RNA Interference

The dsRNA products were prepared as previously described (52). cDNA of *Drosophila IMD*, *MyD88*, *NOS*, and *CanA1* and plasmid GFP- pEASY-T1 (TransGen) were used as templates for PCR amplification using gene-specific primers (**Table S1**). The conditioned medium (1 mL) from S2 cells cultured in 12 well plate was replaced with 0.5 mL of Schneider’s *Drosophila* medium containing 6 µg of dsRNA samples of *IMD*, *MyD88*, *NOS*, *CanA1*, or a mixture of *dsIMD* and *dsMyD88* (6 µg each). After 1 h incubation, 0.5 mL of Schneider’s *Drosophila* medium containing 10% FBS was added to each well. Equal amount of GFP dsRNA was added as a control. RNAi efficiency was examined three days after dsRNA treatment using qPCR as described below. For the RNAi treatment combined with bacterial incubation, the killed bacteria were added to each well at 72 h post dsRNA treatment, and total RNA samples were prepared 24 h later.

### Treatment of S2 Cells With Compounds

Stock solution (250 mM) of diethylamine NONOate (Sigma D184, an NO releasing compound or NOC) was dissolved in water prior to use. To treat S2 cells, the NOC at 2.5 mM final concentration was used to increase NO level in the medium. S2 cell and medium samples were collected at 0, 6, 12, 24, and 48 h after NOC addition. Calcineurin A inhibitor FK506 (Sigma, F4679) was dissolved in DMSO to make a 100 mM stock. FK506 at 0, 10, 20, 30 and 50 mM along with 2.5 mM NOC was used to treat S2 cells and test influence of FK506 on the regulation of AMP production by NO. S2 cell and medium samples were collected at 24 h post NOC-FK506 treatment, PBS was used as control. Stock solution (400 mM) of *N*_ω_-nitro-*L*-arginine methyl ester (Sigma N5751, L-NAME, a NOS inhibitor) was dissolved in water and used at 200 μM along with dead bacteria in the medium as indicated to test the effect of L-NAME on AMP expression. S2 cells and medium samples were collected at 24 h post treatment. PBS and killed bacteria were used as negative and positive controls, respectively.

### qPCR Analysis

S2 cells (1×10^6^) or 3 whole larvae were collected from each biological treatment and replicate. Total RNA was extracted using 1 mL Trizol, RNA concentrations were determined on an Eppendorf BioPhotometer D30, and RNA integrity was examined by 1% agarose gel electrophoresis. cDNA templates were generated from 1 μg total RNA using HiScript III RT SuperMix for qPCR in the presence of genomic DNA wiper (Vazyme, Nanjing, China). Diluted cDNA (1:10, 1 μL) was used for qPCR analysis on a Bio-Rad CFX96 Real Time Detection System (Bio-Rad, CA, United States) in 20 μL reaction containing 1 μL of cDNA, 10 μL of AceQ Universal SYBR qPCR Master Mix (Vazyme), 1.0 μL each of forward and reverse primers (10 μM) and 7 μL ddH_2_O. The thermal cycling conditions were initial denaturation at 95°C for 10 min, followed by 40 cycles of denaturation at 95°C for 10 s and annealing-extension at 60°C for 30 s, with melting curve measured from 60 to 95°C. All the treatments were in triplicate. *O. furnacalis* reference gene ribosomal protein L8 (RPL8) gene (53) and *D. melanogaster* reference gene ribosomal protein 49 (RP49) (54)were used to calibrate the relative expressions of target genes. The mRNA level changes of interested genes were determined using the relative quantitative method (2^−ΔΔCt^) (55). qPCR data were plotted using GraphPad (Version 9.0.2) for statistical analysis. Student’s t-test results are shown as ∗, *p* < 0.05; ∗∗, *p* < 0.01; ∗∗∗, *p* < 0.001. Results of one-way ANOVA followed by Tukey’s test are marked similarly.

### Determination of Nitric Oxide Concentration

To determinate NO concentrations in the media of S2 cells and hemolymph of *O. furnacalis* larvae, the samples were collected by centrifugation at 16,000×*g* for 30 s to remove cells, and the supernatants were used for measuring NO concentrations. The supernatants of medium samples and 1:100 diluted larval plasma (30 μL) were taken to measure NO levels using Total Nitric Oxide Assay Kit (Beyotime, Beijing, China) according to the manufacturer’s instructions (56).

## Results

### Inhibition of NOS and CanA Increased the Mortality of *O. furnacalis* After Bacterial Infection

**Figure 1:**
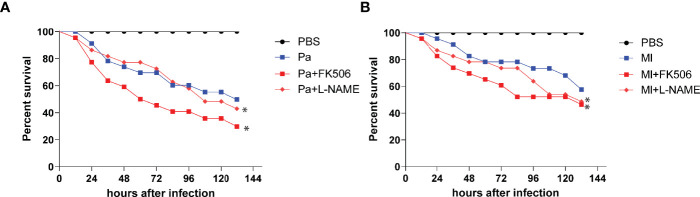
Inhibition of CanA and NOS caused more deaths of infected *O. furnacalis* larvae. After elimination of indigenous bacteria using 50 μg/μL tetracycline, day 3, 4^th^ instar larvae were injected with 1× 10^4^ cells of *P. aeruginosa* **(A)** or *M. luteus* **(B)** cells or along with 2 nmol FK506 or L-NAME, using PBS as control. All the data was analyzed using the log-rank test. *, *p <*0.05; FK506, CanA inhibitor; L-NAME, NOS inhibitor; Pa, *P. aeruginosa*; Ml, *M. luteus*.

To assess the immune stimulatory effect of *P. aeruginosa* and *M. luteus* on *O. furnacalis*, we injected larvae with different numbers of live bacteria and found that the larvae reached 50% mortality after injected with about 1×10^4^ CFUs of *P. aeruginosa* or *M. luteus* (**Figure S1**). Thus, 1×10^4^ CFUs of these two bacteria were used to challenge *O. furnacalis* larvae in later experiments. Injection of the NOS or CanA inhibitor caused higher mortality of larvae upon bacterial challenge (**Figure 1**), suggesting an involvement of NOS and CanA in the immune responses to bacterial infection in *O. furnacalis*.

### Induction of NOS, CanA and AMPs by Bacterial Infection in *O. furnacalis* Larvae

**Figure 2:**
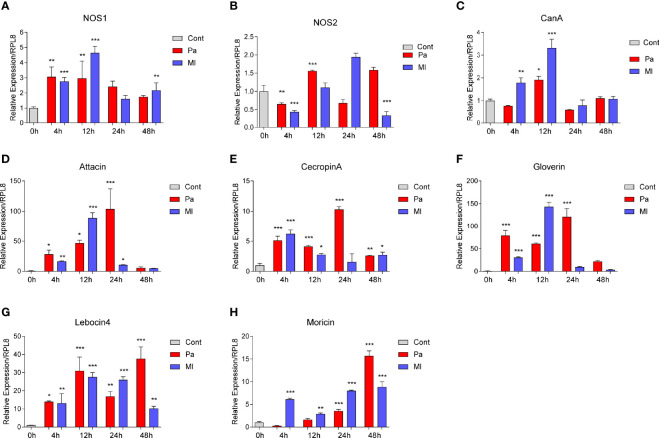
Expression changes of *NOS*, *CanA* and *AMPs* in *O. furnacalis* larvae after bacterial infection. mRNA level changes in *NOS* **(A, B)**, *CanA* **(C)**, *Attacin* **(D)**, *Cecropin A* **(E)**, *Gloverin* **(F)**, *Lebocin4* **(G)**, and *Moricin* **(H)** in whole *O. furnacalis* larvae at certain times post 1×10^4^ cells of *P. aeruginosa* or *M. luteus* infection. Cont, PBS as control; CanA, calcineurin A; NOS, nitric oxide synthase; Pa, *P. aeruginosa*; Ml, *M. luteus*. One-way ANOVA analysis followed by Tukey’s test was used to compare the control and infected groups. *p < 0.05; **p < 0.01; ***p < 0.001.

NO and AMPs are effectors that eliminate invading bacteria in insects, and some research indicated cross-talks between ROS and AMP production (47). To investigate whether or not NOS and CanA are involved in the processes in *O. furnacalis* larvae, we first measured the transcript levels of *NOS*, *CanA* and *AMPs* under immune stress (**Figure 2**). *NOS1* but not *NOS2* mRNA level was strongly induced (**Figures 2A, B**), and the expression levels of *NOS1* and *NOS2* in different tissues showed that *NOS1* was mainly expressed in hemocytes, while *NOS2* were mainly expressed in fat body (**Figure S5**). *NOS1* and *CanA* showed a similar expression pattern, which were mainly upregulated at 4 and 12 h post infection (**Figures 2A, C**). In addition, we found that *CanA* was also mainly expressed in hemocytes (**Figure S5**). The AMP effector genes were upregulated following the increase of *NOS1* and *CanA* expression (**Figures 2D−H**). These data provided clues for us to explore the mechanism for NO-regulated AMP expression during immune responses.

### NO Increased mRNA Levels of CanA and Some AMPs in *O. furnacalis* Larvae

**Figure 3:**
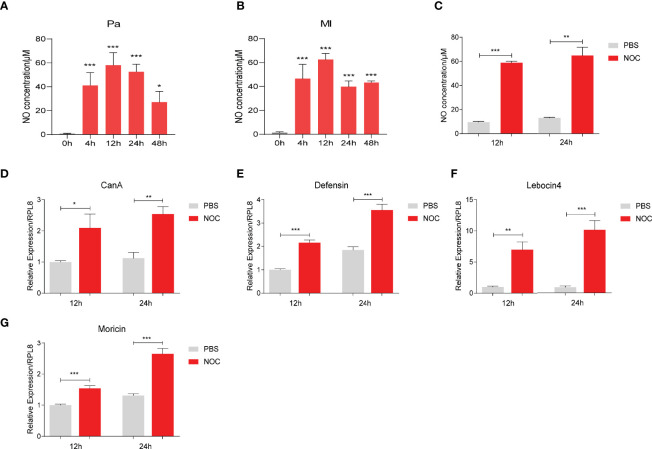
NO increased CanA and AMP expression in *O. furnacalis* larvae. NO concentrations in hemolymph after infection by 1×10^4^ CFUs of *P. aeruginosa* **(A)** and *M. luteus* **(B)** at 4 to 48 h post injection. NO concentrations in larval hemolymph after injection of the NOC **(C)**. Transcript levels of *CanA* **(D)**, *Defensin* **(E)**, *Lebocin* 4 **(F)** and *Moricin* **(G)** after NOC injection. One-way ANOVA followed by Tukey’s test was used to compare the control and infected groups **(A, B)**. Student’s t-test was used to compare PBS- and NOC-treated groups **(C−G)**. *p < 0.05; **p < 0.01; ***p < 0.001.

NOS catalyzes the production of NO from an endogenous substrate L-arginine. After infection with *P. aeruginosa* or *M. luteus*, NO concentrations in hemolymph increased significantly at 4, 12, 24 and 48 h (**Figures 3A, B**). Injection the diethylamine NONOate (NOC, an NO donor) also increased NO concentration to a similar level in hemolymph (**Figure 3C**). NO also induced the expression of *CanA*, and *Defensin*, *Lebocin4* and *Moricin* (**Figures 3D−G**). However, NOC did not induce *Attacin*, *CecropinA* or *Gloverin* expression (**Figure S2**), suggesting NO has some specificity in inducing AMP production. Thus, we hypothesized that CanA may participate in bacteria-induced NO production to regulate the expression of certain AMPs.

### *E. coli* and NO Releasing Compound (NOC) Increased NO Concentration in the Medium of S2 Cells

**Figure 4:**
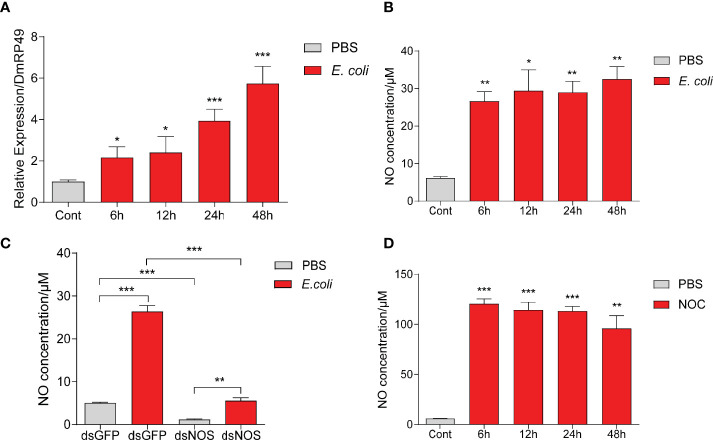
*E coli* and NOC treatments stimulated NO production in *Drosophila* S2 cells. NOS mRNA levels **(A)** and NO concentrations **(B)** after *E coli* infection. NO levels in the medium samples after *NOS* RNAi and treatment with killed *E coli* **(C)** or the NOC **(D)**. Cont, PBS treatment at 0 h One-way ANOVA followed by Tukey’s test was used to compare control and treatment groups **(A, B, D)**. Student’s t-test was used to analyze significance in **(C)**. *p < 0.05; **p < 0.01; ***p < 0.001.

To understand how NO may regulate AMP production, we used *Drosophila* S2 cells in further tests. Incubation with dead *M. luteus* and *E. coli* induced S2 cells to make AMPs (**Figure S3**). *E. coli* from 1 mL culture at OD_600_ = 1.0 led to a stronger AMP response than the Gram-positive bacteria. Thus, this amount of dead *E. coli* was chosen to treat S2 cells in the later experiments. We found that the *NOS* expression and NO production were strongly induced by *E. coli*, as in *O. furnacalis* larvae (**Figures 4A, B**, **2A**). Knockdown of *NOS* reduced the NO level increased by *E. coli* (**Figure 4C**). After incubation with the NOC, NO concentration in the cell culture medium increased and lasted for two days at least (**Figure 4D**). Therefore, *Drosophila* S2 cells appear to be a good model for investigating the link between NO and AMP production.

### Induction of AMPs by NO in S2 Cells

**Figure 5:**
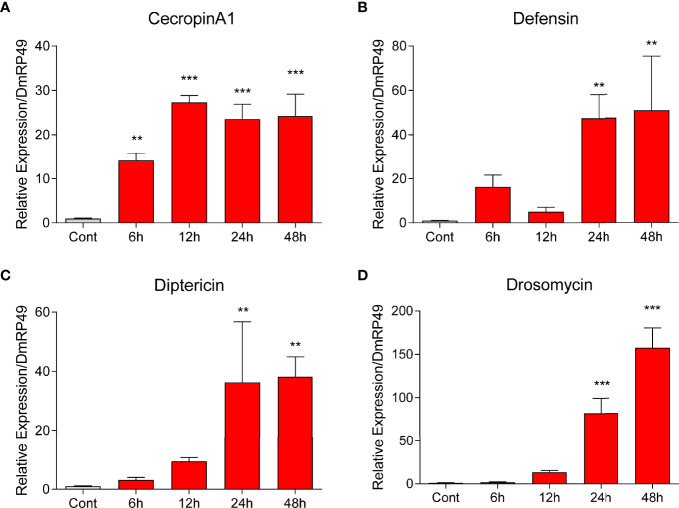
NOC induced AMP expression in *Drosophila* S2 cells. The NOC at a final concentration of 2.5 mM was used to treat S2 cells. Transcript levels of the four AMPs *Cecropin A1* **(A)**, *Defensin* **(B)**, *Diptericin* **(C)**, and *Drosomycin* **(D)** at different time points were measured by qPCR. One-way ANOVA followed by Tukey’s test was used to compare control and treated groups. **p < 0.01; ***p < 0.001.

To further uncover the role of NO in AMP production, we added NOC (NO donor) to the culture of S2 cells. At 24 and 48 h, the transcript levels of *CecropinA1*, *Defensin*, *Diptericin* and *Drosomycin* increased significantly (**Figure 5**). The effect was observed for *CecropinA1* at 6 and 12 h, suggestive of a more sensitive response to NO for this gene. NO concentration elevations caused by *E. coli* or the NOC were detected at 6, 12, 24, and 48 h (**Figures 4B, D**). The major induction of AMPs at 24 and 48 h suggested that the NO involvement in AMP production may be indirect, relying on protein products of intermediate gene(s).

### IMD Pathway Connected the NO Signal to AMP Production in S2 Cells

**Figure 6:**
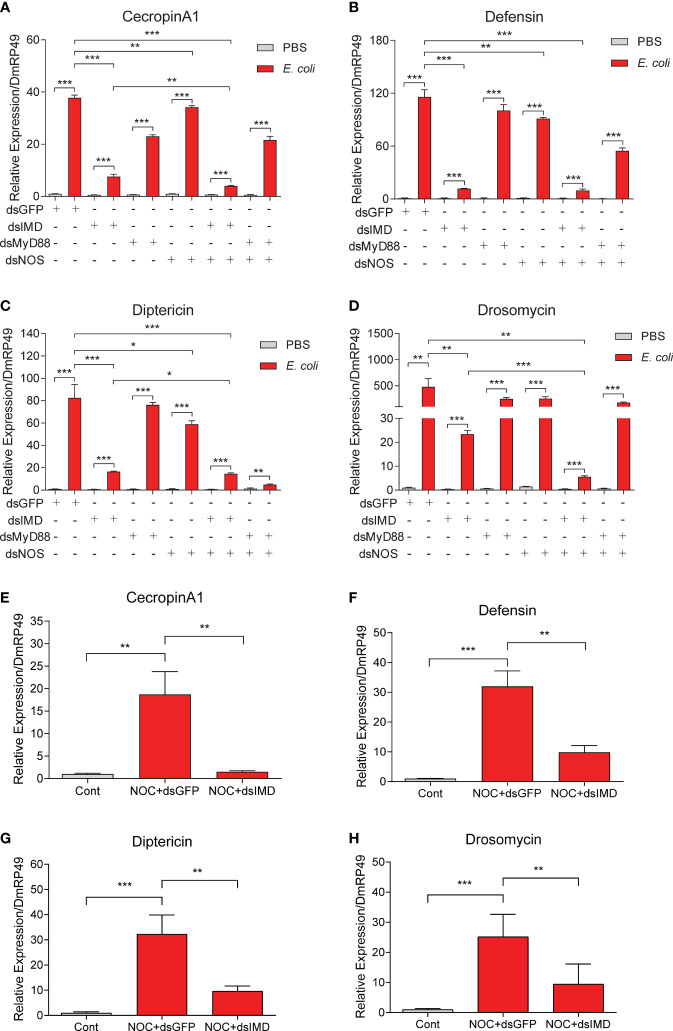
Effects of *IMD*, *MyD88* or *NOS* knockdown on AMP expression in *Drosophila* S2 cells. To analyze possible roles of Toll/IMD pathway and *NOS* in NO-induced AMP expression, RNAi of *IMD*, *MyD88* or *NOS* was performed in S2 cells for 48 h in advance of the treatment by killed *E coli*. Transcript levels of *Cecropin A1* **(A)**, *Defensin* **(B)**, *Diptericin* **(C)** and *Drosomycin* **(D)** were analyzed by qPCR. The effects of IMD knockdown on induction of *CecropinA1* **(E)**, *Defensin* **(F)**, *Diptericin* **(G)** and *Drosomycin* **(H)** by the NOC were detected. Student’s t-test was used to analyze significance, *p < 0.05; **p < 0.01; ***p < 0.001.

Since AMP expression is known to be controlled by the Toll and IMD pathways (14, 37, 38, 57), how may NO-induced AMP production in S2 cells (**Figure 5**) and *O. furnacalis* larvae (**Figure 3**) be linked to the two classic pathways? To address this question, we employed RNA interference to knockdown the pathway components and determine whether the NO-induced AMP production is affected in S2 cells. We found the increases in mRNA levels of *CecropinA1*, *Defensin*, *Diptericin* and *Drosomycin* caused by dead *E. coli* were dramatically suppressed after *IMD* had been knocked down (**Figures 6,** **S4**). Treatment with dsRNA of *MyD88* had a lesser effect. Therefore, NO-induced AMP production was regulated mainly by the IMD pathway but not much by Toll signaling. Similarly, the AMP transcription increases were partly suppressed by *NOS* dsRNA, suggesting the NOS may take part in the AMP induction upon *E. coli* treatment. Furthermore, knockdown of *IMD* in S2 cells significantly reduced *CecropinA1*, *Defensin*, *Diptericin* and *Drosomycin* expression, which induced by NOC treatment (**Figures 6E–H**). Together, these data suggested that NO is involved in the upregulation of *CecropinA1*, *Defensin*, *Diptericin* and *Drosomycin* transcription through the IMD pathway.

### NOS Was Required for the Upregulation of CanA1 in S2 Cells Induced by *E. coli*

**Figure 7:**
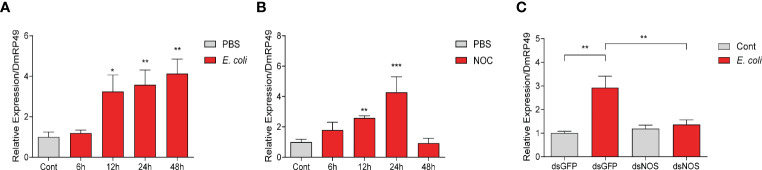
NO contributed to the upregulation of *CanA1* induced by killed *E coli* in *Drosophila* S2 cells. The expression pattern of *CanA1* in response to *E coli* treatment **(A)**, NO donor **(B)**, and *NOS* knockdown followed by *E coli* treatment **(C)** was determined by qPCR. Cont: PBS treatment; NOC: Nitric oxide donor. One-way ANOVA followed by Tukey’s test was used to compare control and treated groups **(A, B)**. Student’s t-test was used to analyze significance **(C)**, *p < 0.05; **p < 0.01; ***p < 0.001.

In *O. furnacalis* larvae, bacterial infections increased the transcript levels of *CanA* and *AMPs*, whereas inhibition of CanA reduced resistance to the infections and resulted in more death (**Figures 1**, **2C**), suggesting the involvement of CanA in the resistance to bacterial infection. To investigate whether CanA is involved in the resistance through regulating the expression of AMPs, S2 cells were used for further studies. We found that dead *E. coli* induced the expression of *CanA1* at 12, 24, and 48 h in S2 cells (**Figure 7A**), and that addition of NO donor NOC also induced the expression of *CanA1* at 12 and 24 h (**Figure 7B**), which indicates that bacterial infection and NO increased expression of *CanA1*. However, knockdown of *NOS* suppressed the induction of *CanA1* by *E. coli* (**Figure 7C**), suggesting that CanA1 might be downstream of NO signal.

### Knockdown and Inhibition of CanA1 Can Block the Upregulation of AMPs by NO in S2 Cells

**Figure 8:**
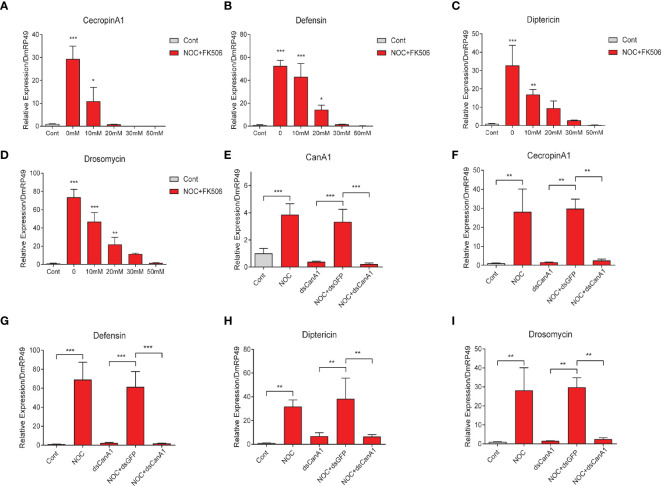
Effects of *CanA1* knockdown and CanA1 inhibitor on NOC-induced AMP expression. Transcript level changes of Cecropin A **(A)**, Defensin **(B)**, Diptericin **(C)** and Drosomycin **(D)** in NOC treated S2 cells after different concentrations of CanA1 inhibitor treatment were determined by qPCR, The concentrations of FK506 were indicated. dsRNA of *CanA1* was used to evaluate the effects of NOC on the expression of *CanA1* **(E)**, *Cecropin A1* **(F)**, *Defensin* **(G)**, *Diptericin* **(H)** and *Drosomycin* **(I)** in response to NOC challenge. Cont, PBS used as control; NOC, nitric oxide donor; CanA1, CalcineurinA1. One-way ANOVA followed by Tukey’s test was used to compare control and treatment groups **(A-D)**. Student’s t-test was used to analyze significance **(E-I)**, *p < 0.05; **p < 0.01; ***p < 0.001.

To further confirm the relationship between CanA1 and NO on controlling AMPs expression in S2 cells, we used CanA1 inhibitor and knockdown to inhibit the activity of CanA1 and reduce the transcript level of *CanA1*, respectively. We found that CanA1 inhibitor could significantly block the expression of *CecropinA1*, *Defensin*, *Diptericin* and *Drosomycin*, which were induced by NO (**Figures 8A–D**). In addition, knockdown of *CanA1* in S2 cells also decreased the expression of *CanA1* induced by the NOC (**Figure 8E**), and significantly suppressed the upregulation of *CecropinA1*, *Defensin*, *Diptericin* and *Drosomycin* by NO (**Figures 8F–I**). These results directly indicated that the AMP expression induced by NO was mediated by CanA1.

## Discussion

After insect innate immunity was discovered, the robust defense system has been well investigated in different species (8, 58). In particular, the immune signaling pathways such as ROS reaction, Toll and IMD pathways, and PPO cascade are well studied (14, 37, 38, 58–60). As a member of the ROS family, NO induced by pathogen infections participates in the regulation of AMP production, mainly regulated *via* classic Toll and IMD pathways. That means that there are cross-talks between the ROS reaction and AMP signaling pathway. In this study, we used a lepidopteran pest *O. furnacalis* to analyze the regulatory mechanism of NO on AMP production and found that bacterial infection can upregulate the expression of *NOS*, *CanA* and certain *AMPs* through NO production. NO and CanA are needed to fight against bacterial infection in *O. furnacalis.* Using S2 cells, we confirmed that CanA1 mediated the regulation of AMP productions between NO signal and IMD pathway. Our work suggests that NO signal might play as the messenger between rapid ROS reaction and AMP signaling pathway.

The mechanism of how CanA regulates the AMPs expression is still unclear. As a member of the protein phosphatase 2B family, calcineurin is a Ca^2+^-dependent phosphatase, involved in many physiological processes such as regulation of Ca^2+^ homeostasis, transcription, and innate immunity (61–63). Calcineurin comprises a catalytic subunit A and a regulatory subunit B (41). In *Drosophila*, there are three catalytic subunits including calcineurin A1, protein phosphatase 2B-14D and calcineurin A-14F (64). Protein phosphatase 2B-14D and calcineurin A-14F can respond to Gram-positive bacterial infection and activate Dorsal to induce AMPs production (41). While calcineurin A1 has effects on regulation of AMPs production *via* Relish in response to Gram-negative bacterial infections or NO challenge (41, 46). Subunit calcineurin A1 can directly receive the signal of NO and act on Relish without the components of IMD pathway, subunits protein phosphatase 2B-14D and calcineurin A-14F directly activate Dorsal/Dif activity dependent on the calcium level altered by Gram-positive bacterial infections (41), suggesting that the regulation of AMPs production by calcineurin subunit A is directly mediated by NF-κB and independent of Toll/IMD pathways. In contrast, the NO-induced AMPs production is dependent on Toll/IMD pathway in *S. exigua* (25). In this study, our results indicate that IMD pathway is required for calcineurin A to mediate the regulation of AMPs by NO. These differences between non-NO and NO mediated AMPs production *via* the CanA1 regulation remain to be fully deciphered in the future. In *B. mori* and *D. melanogaster*, eicosanoids are involved in AMPs production (65, 66). Inhibition of phospholipase A2 (PLA2) activity can reduce the biosynthesis of eicosanoids, and finally decreases AMPs production in *S. exigua* (67). In addition, NO increased the activity of PLA2, and PLA2 was capable to upregulate the AMP production *via* eicosanoids in *S. exigua* (25, 68). Therefore, whether or not calcineurin A can regulate the PLA2 to alter AMPs production *via* eicosanoids need further investigation.

**Figure 9:**
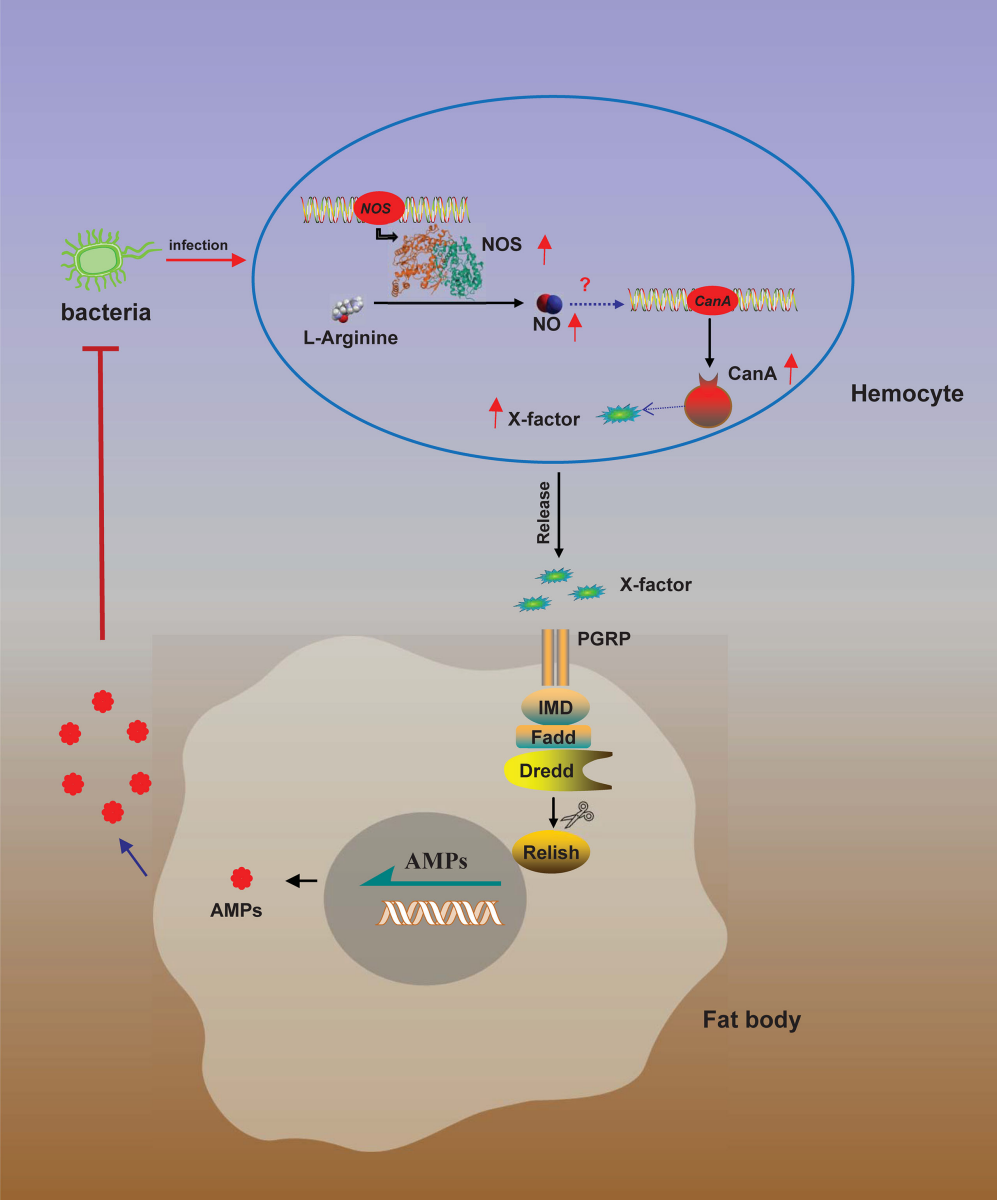
A model for NO-regulated AMP production. Bacterial infection induces *NOS* expression in hemocytes to convert L-arginine to NO by NOS. NO then induces CanA expression to produce and release of an unknown factor (X) from hemocytes. Released X activates *AMP* expression in fat body *via* IMD pathway to eliminate the invading bacteria.

There is organ-to-organ communication during immune responses. In the *Drosophila* gut, the ROS reaction and IMD pathway producing AMPs play primary roles in the elimination of gut microbes (8). Enterobacteria *Ecc15* oral infection can locally trigger the expression of AMPs and ROS reaction in adult *Drosophila* gut (4), although *Ecc15* can’t cross through the gut and enter the hemolymph, the local infection in the gut also upregulates *AMPs* expression in fat body (69, 70). Furthermore, ROS stress induced by local *Ecc15* oral infection in *Drosophila* gut upregulates the production of NO in gut, and then the NO signal as a messenger is relayed by hemocytes to trigger the expression of AMP *Diptericin* in the remote organ fat body (15). In this study, we found that *NOS1* in hemocytes was the primary NOS in response to bacterial infections in *O. furnacalis* (**Figures 2**, **S5**), while AMPs were mainly expressed in gut and fat body (8, 25), we inferred that there might be also a link between hemocyte producing NO and fat body expressing AMPs in *O. furnacalis*. Based on the tissue expression analysis of *CanA*, *NOS1* and *NOS2*, and the responses of these three genes to bacterial infections in *O. furnacalis*, we inferred NO production induced by bacteria in hemocytes increased the expression of *CanA* in hemocytes together with some unknown factors, which were released from hemocytes to induce *AMPs* expression in fat body *via* IMD pathway (**Figure 9**). To further confirm the organ-to-organ immune signals in *O. furnacalis*, *Ex vivo* assay using hemocytes and fat body from *O. furnacalis* larvae might need to be set up in the future. Using condition medium collected from dead bacteria-challenged hemocytes to stimulate the germ-free fat body may be an ideal approach to investigate the organ-to-organ communication and identify the unknown factors from hemocytes to fat body.

NO plays a minor role in bacteria-induced AMP production. The classic pathways regulating AMP productions are Toll and IMD pathways in several model insects (71, 72). Toll pathway and IMD pathway are activated by Lys-peptidoglycans (from Gram-positive bacteria) and DAP-peptidoglycans (DAP: meso-diaminopimelic acid, mainly from Gram-negative bacteria), respectively (73). In this study, different amounts of killed Gram-positive and Gram-negative bacteria *M. luteus* and *E. coli* were used to treat S2 cells, even a small amount of *E. coli* showed stronger activity than *M. luteus* to induce AMPs expression in S2 cells. We found that knockdown of *NOS* did not totally block the induction of AMPs after bacterial infection, and NO only induced considerable AMPs, indicating that the AMPs production induced by NO takes part in the AMP production through IMD pathway in S2 cells.

So far, the mechanism of NO production after bacterial infection is unclear. In *S. exigua*, the expression of AMPs is under the regulation of Toll pathway and IMD pathway like that in other insects (14, 25, 74, 75). NO also can regulate the expression of AMPs in *S. exigua*, while knockdown of *Toll* or *Relish* decreases the expression of *NOS*, and reduces NO concentration in response to bacterial infection, suggesting that the NO signal is downstream of IMD and Toll pathway in *S. exigua* (25). In *Drosophila*, the NO-induced AMPs production is independent of Toll and IMD pathways (41). In this study, our result showed that NO upregulated the expression of *CanA1*, and then CanA1 activated the expression of *AMPs via* IMD pathway in S2 cells. Knockdown of both *NOS* and *IMD* could block expression of *AMPs* similar as knockdown of *IMD* only did, suggesting that the NO signaled IMD pathway to regulate the *AMP* expression.

## Conclusion

In our study, we found that NO donor could induce the expression of *Cecropin A*, *Defensin*, *Diptericin* and *Drosomycin* in S2 cells (**Figure 5**), while in *O. furnacalis* larvae, NO donor significantly induced the expression of *Defensin*, *Lebocin4* and *Moricin* but not *Attacin*, *Cecropin A1* and *Gloverin* (**Figures 3**, **S1**). NO donor can upregulate the expression of *Attacin1/2*, *Defensin* and *Gloverin* in *S. exigua* (25). We also used *B. mori* to analyze the AMPs expression after bacterial infection or NO donor treatment (**Figure S6A**), and found that NO donor NOC had strong activity to induce the expression of AMPs *Cecropin D*, *Cecropin E*, *Lebocin*, *Moricin* and *Defensin A*. Moreover, inhibition of NOS using L-NAME increased the death of bacterial infected *B. mori* larvae (**Figure S6B**), which was consistent with the survival assay using *O. furnacalis* larvae. These data indicated that NO can specifically induce certain AMPs in different insects.

## Data Availability Statement

The original contributions presented in the study are included in the article/**Supplementary Material**. Further inquiries can be directed to the corresponding authors.

## Author Contributions

CF, KC and ZL designed the study. XWa, JC and XWe performed the experiments. KC and JC performed statistical analysis, KC prepared the first draft. CF, HJ, ZL and YW revised and finalized the manuscript. All authors contributed to the article and approved the submitted version.

## Funding

This work was supported by National Natural Science Foundation of China (31901876, 31871952, and 31970467), Natural Science Foundation of Jiangsu Province (KB20190900), China Postdoctoral Science Foundation (2018M642343) and NIH grant GM58634.

## Conflict of Interest

The authors declare that the research was conducted in the absence of any commercial or financial relationships that could be construed as a potential conflict of interest.

## Publisher’s Note

All claims expressed in this article are solely those of the authors and do not necessarily represent those of their affiliated organizations, or those of the publisher, the editors and the reviewers. Any product that may be evaluated in this article, or claim that may be made by its manufacturer, is not guaranteed or endorsed by the publisher.

## Supplementary Material

Supplementary Table 1 Primers used in this study.

Supplementary Figure 1 Survival of *O. furnacalis* larvae after bacterial infection. Survival curves of the larvae after an exposure to live *P. aeruginosa* **(A)** and *M. luteus* **(B)** at different dosages (1×10^3^ to 1×10^6^ CFUs/larva)

Supplementary Figure 2 NOC did not induce *Attacin*, *Cecropin A* or *Gloverin* synthesis in *O. furnacalis* larvae.

Supplementary Figure 3 Induced transcription of *Cecropin A1* **(A, B)**, *Diptericin* **(C, D)**, *Defensin* **(E, F)**, and *Drosomycin* **(G, H)** in *Drosophila* S2 cells by different amounts of killed *M. luteus* **(A, C, E, G)** and *E. coli* **(B, D, F, H)**.

Supplementary Figure 4 The efficiency of RNAi against the target genes in *Drosophila* S2 cells.

Supplementary Figure 5 Expression of *CanA*, *NOS1* and *NOS2* genes in different tissues of *O. furnacalis* larvae. Different letters above a given treatment indicate means significantly differ. One-way ANOVA followed by Tukey’s test was used to compare each other.

Supplementary Figure 6 Effects of NO donor and bacterial infections on *AMP* expression **(A)** in and survival curves **(B)** of *B. mori* larvae. Day 3, 5^th^ instar *B. mori* larvae were injected with NO donor NOC or S-nitroso-N-acetylpenicillamine (SNAP) (20 nmol/larva), 1×10^7^ cells of *P. aeruginosa* or *S. aureus*, or 1×10^7^ cells of *P. aeruginosa* or *S. aureus* together with NOS inhibitor L-NAME (2 nmol/larva). Fat body was collected at 6 h post infection, and transcript level change of *Cecropin A*, *Cecropin B*, *Cecropin D*, *Cecropin E*, *Gloverin 4*, *Lebocin*, *Moricin* and *Defensin A* were determined by PCR **(A)**. The survival curve was also plotted after bacterial infection with or without NOS inhibitor L-NAME **(B)**.
