# Supplementary figures and images for: Nitric Oxide-Induced Calcineurin A Mediates Antimicrobial Peptide Production Through the IMD Pathway
Source: Front Immunol. 2022 May 18;13:905419. doi: 10.3389/fimmu.2022.905419 (PMC9157438; doi:10.3389/fimmu.2022.905419)

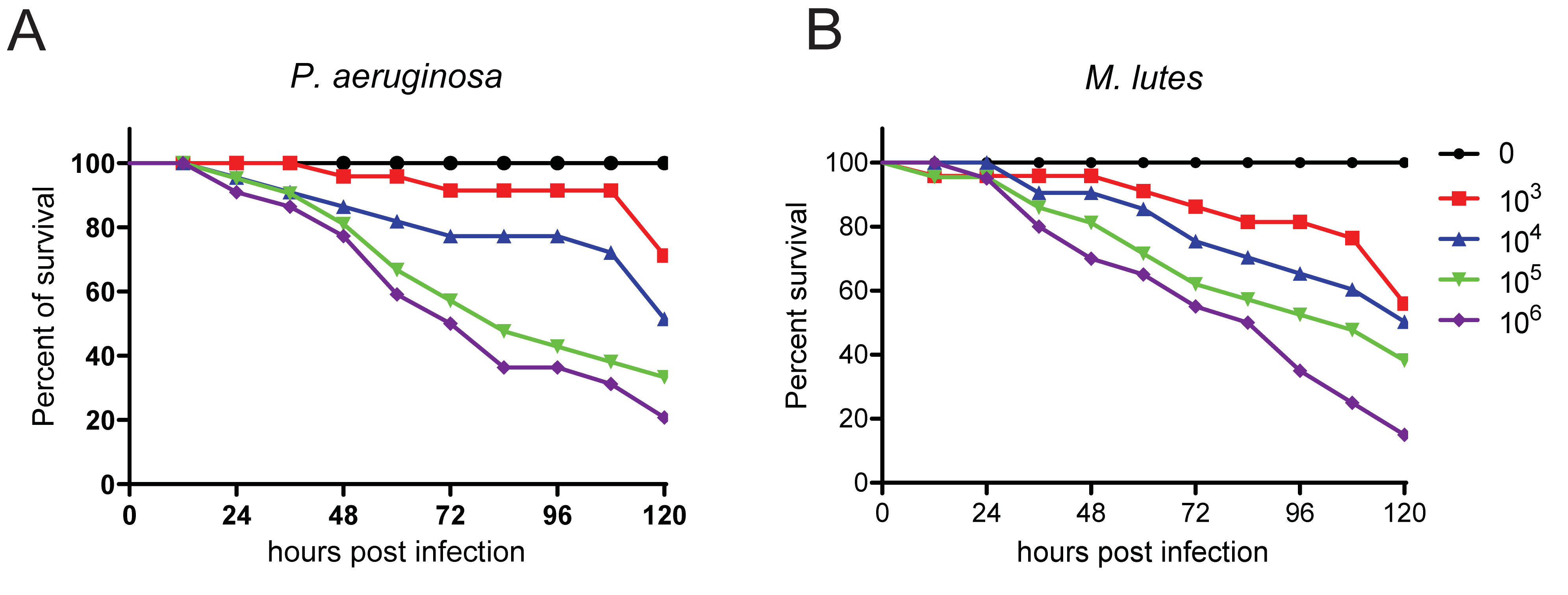

Supplement: Supplementary Figure 1 — Survival of O. furnacalis larvae after bacterial infection. Survival curves of the larvae after an exposure to live P. aeruginosa (A) and M. luteus (B) at different dosages (1×10^3^ to 1×10^6^ CFUs/larva) [file Image_1.tif]

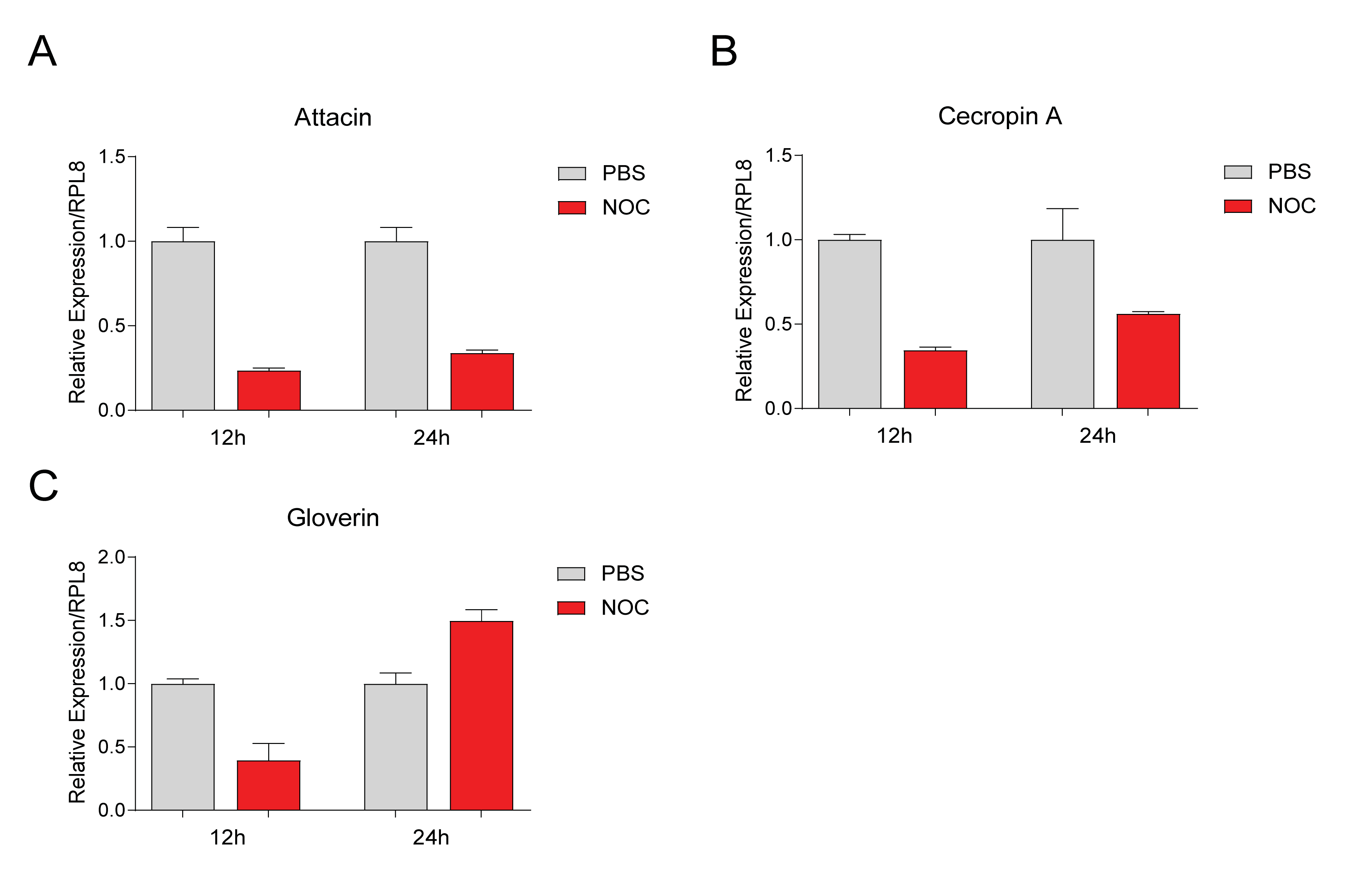

Supplement: Supplementary Figure 2 — NOC did not induce Attacin, Cecropin A or Gloverin synthesis in O. furnacalis larvae. [file Image_2.tif]

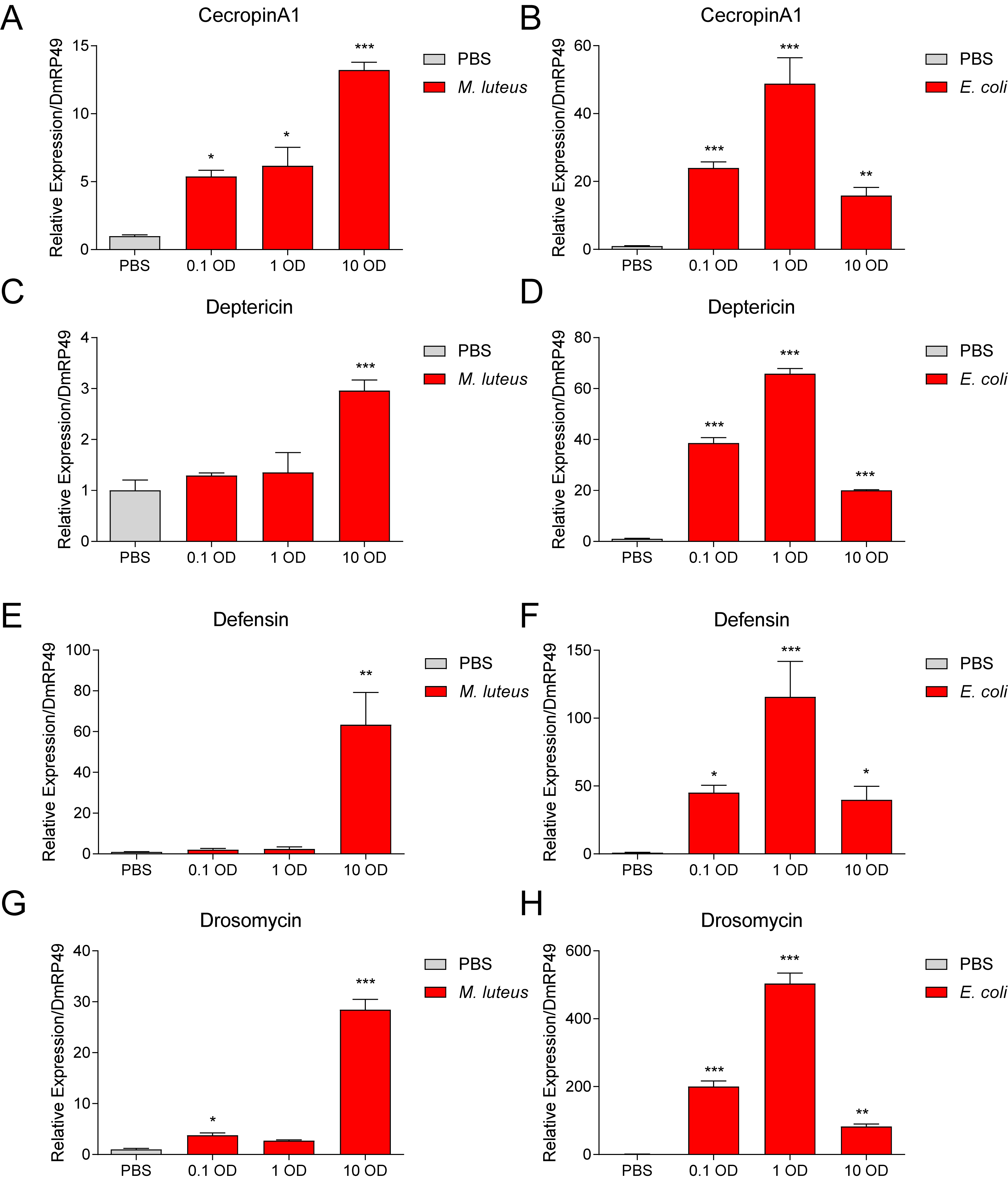

Supplement: Supplementary Figure 3 — Induced transcription of Cecropin A1 (A, B), Diptericin (C, D), Defensin (E, F), and Drosomycin (G, H) in Drosophila S2 cells by different amounts of killed M. luteus (A, C, E, G) and E. coli (B, D, F, H). [file Image_3.tif]

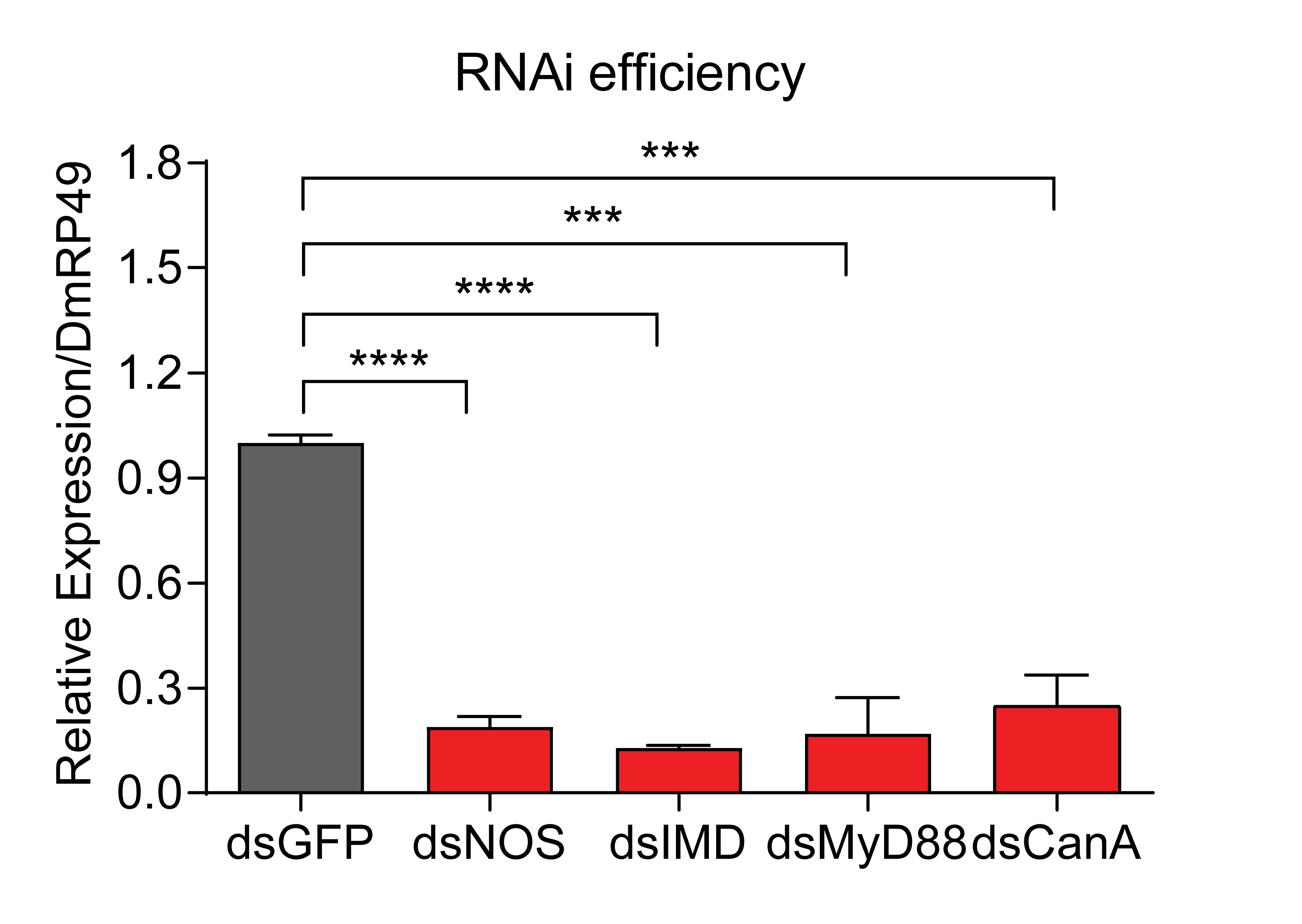

Supplement: Supplementary Figure 4 — The efficiency of RNAi against the target genes in Drosophila S2 cells. [file Image_4.tif]

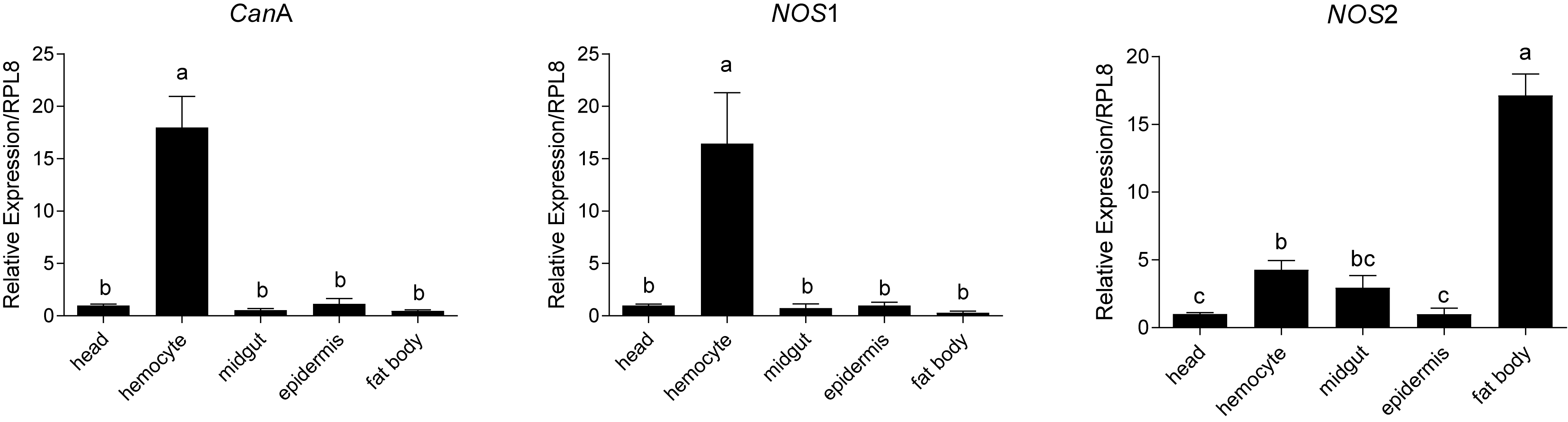

Supplement: Supplementary Figure 5 — Expression of CanA, NOS1 and NOS2 genes in different tissues of O. furnacalis larvae. Different letters above a given treatment indicate means significantly differ. One-way ANOVA followed by Tukey’s test was used to compare each other. [file Image_5.tif]

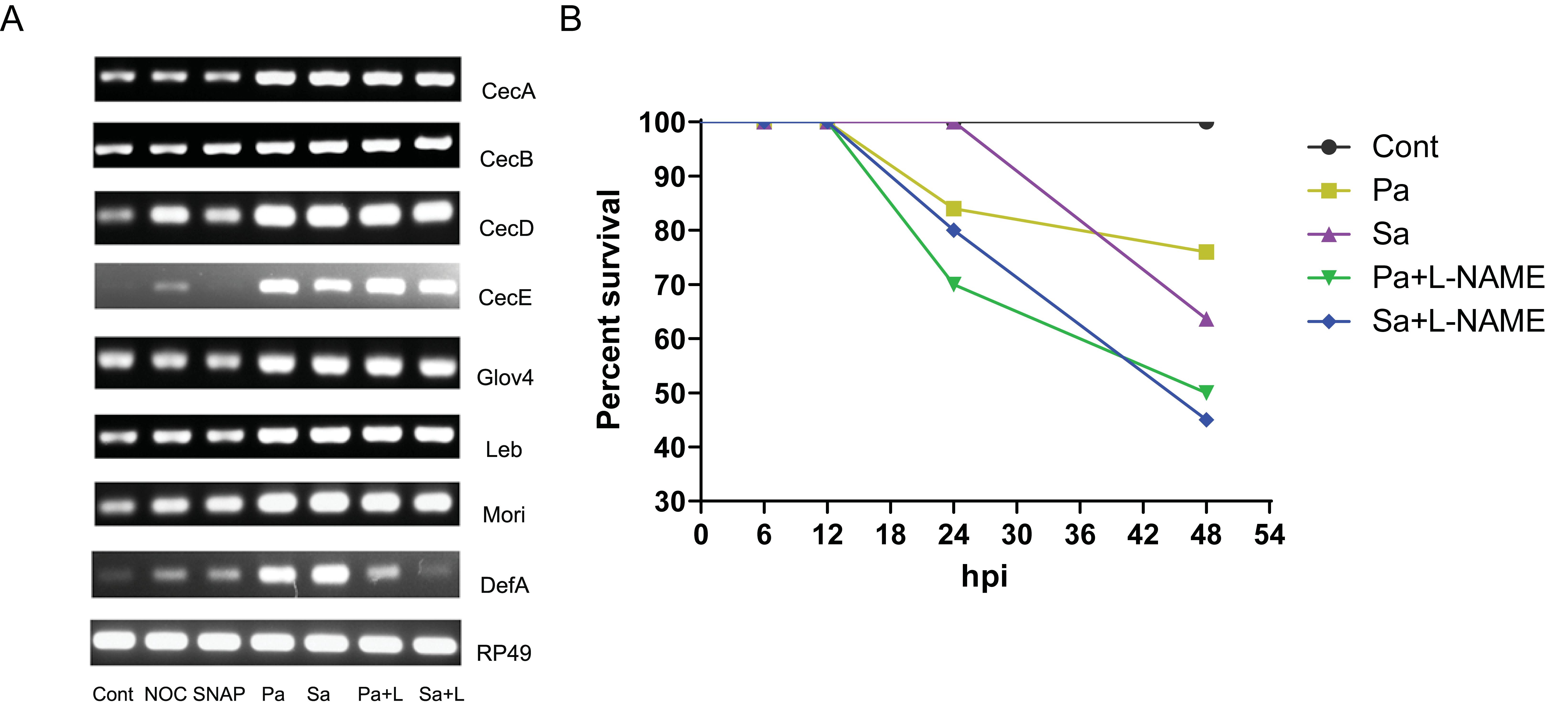

Supplement: Supplementary Figure 6 — Effects of NO donor and bacterial infections on AMP expression (A) in and survival curves (B) of B. mori larvae. Day 3, 5th instar B. mori larvae were injected with NO donor NOC or S-nitroso-N-acetylpenicillamine (SNAP) (20 nmol/larva), 1×10^7^ cells of P. aeruginosa or S. aureus, or 1×10^7^ cells of P. aeruginosa or S. aureus together with NOS inhibitor L-NAME (2 nmol/larva). Fat body was collected at 6 h post infection, and transcript level change of Cecropin A, Cecropin B, Cecropin D, Cecropin E, Gloverin 4, Lebocin, Moricin and Defensin A were determined by PCR (A). The survival curve was also plotted after bacterial infection with or without NOS inhibitor L-NAME (B). [file Image_6.tif]
